# Short-Term Clinical and Biochemical Outcomes of Infants Born After 34 Weeks of Gestation with Mild-to-Moderate Cord Blood Acidosis—A Retrospective Study

**DOI:** 10.3390/jcm14165720

**Published:** 2025-08-13

**Authors:** Ayala Gover, Arieh Riskin, Livnat Sharkansky, Rawan Hijazi, Ranin Ghannam, Hussein Zaitoon

**Affiliations:** 1 Ruth and Bruce Rappaport Faculty of Medicine, Technion-Israel Institute of Technology, Haifa 3525433, Israel; arik.riskin@gmail.com (A.R.); hussein.zaitoon@gmail.com (H.Z.); 2 Department of Neonatology and Neonatal Intensive Care, Bnai Zion Medical Center, Haifa 3339419, Israel; 3 Department of Pediatrics, Bnai Zion Medical Center, Haifa 3339419, Israel

**Keywords:** acidosis, umbilical cord blood, base excess, pH

## Abstract

**Background/Objectives**: Umbilical cord pH is used as a predictor of risk for poor neurologic outcome in high-risk newborns. While data on neonates with severe acidemia show a strong association with birth asphyxia and long-term adverse outcomes, the significance of mild-to-moderate acidemia is less clear. This study aimed to investigate short-term outcomes of late preterm and term infants born with mild-to-moderate cord blood acidosis and to compare the predictive ability of pH and base excess. **Methods**: This was a retrospective cohort study, including term and late preterm (≥34 weeks) neonates with mild–moderate umbilical cord blood acidosis, defined as pH 7.0–7.2 or base excess between −12 and −16. Data including demographic, clinical, and laboratory measures were extracted. The cohort population was stratified based on the level of acidosis, mild or moderate, with separate analyses performed by definitions of pH and BE. Mild acidosis was defined as cord blood pH 7.13–7.20 and base excess > −12 and moderate acidosis was defined as cord blood pH 7.00–7.12 or base excess between −12 and −16. **Results**: The study included 337 newborns. Most had mild acidosis, but 90 infants had BE of −12 to −16 and 86 infants had pH 7.00–7.12. Rate of NICU admission was 12.8% (43/337), rising up to 27% among newborns with moderate acidosis. The leading cause for admission was respiratory distress, and there were no cases of moderate–severe HIE. Renal and hepatic dysfunction were more common in moderate compared to mild acidosis; however, most lab abnormalities were mild and transient. Using ROC curves, BE ≤ −12 was found to be a better predictor for renal and liver involvement than pH ≤ 7.12. **Conclusions**: Moderate acidosis in cord blood was associated with an increased incidence of short-term neonatal morbidities, NICU admissions and renal or liver dysfunction compared to mild cord blood acidemia. BE correlated with abnormal values better than pH. Infants with cord gas BE levels ≤ −12 may benefit from closer clinical monitoring and assessment of renal and liver function.

## 1. Introduction

Normal birth involves transient falls in oxygen delivery to the fetus during uterine contractions. This intermittent hypoxemia is well tolerated under normal conditions due to physiological protective mechanisms. However, when O_2_ deprivation is prolonged, anaerobic metabolism ensues with the production of H^+^, resulting in metabolic acidosis. Thus, umbilical cord pH is a proxy for the level of fetal oxygenation, and reflects the condition of the fetus at the time of birth [1]. As such, umbilical cord blood gas is used as a predictor of risk for poor neurologic outcome in high-risk newborns [2,3] and is widely accepted as a valuable tool for assessing newborn status at birth [1]. Following the British National Institute for Health and Care Excellence (NICE) and the American College of Obstetrics & Gynecology (ACOG) recommendations, umbilical blood gas is typically obtained in every high-risk pregnancy and delivery when there are intrapartum signs of fetal distress, such as non-reassuring fetal heart rate (FHR), and in instrumental deliveries [4,5]. Some units extend this practice to perform universal sampling in all deliveries [2,6].

Umbilical cord pH is influenced by various factors [7,8,9,10,11]. Infants born by elective cesarean section without labor have pH values similar to adult values [7]. Lower pH values are observed in infants undergoing normal vaginal delivery and twin labor [8]. Maternal regional anesthesia, particularly spinal, can increase the incidence of cord blood acidosis [9]. Umbilical cord morphology has uncertain clinical importance, and the presence of true knotting of the cord is rare and seldom problematic [10,11]. In several large studies, mean umbilical artery pH in newborns ranged from 7.24 to 7.28, and venous umbilical pH ranged from 7.32 to 7.34 [1]. Different cutoffs for abnormal cord pH have been used in the past [1]. Umbilical artery pH level below 7.0 is common in infants with hypoxic-ischemic encephalopathy (HIE) who present with altered consciousness, abnormal tone and reflexes and multi-organ dysfunction [3]. These infants are at increased risk of severe short-term morbidity including seizures, respiratory distress, mechanical ventilation, metabolic abnormalities and hepatic and renal dysfunction, as well as long-term complications such as seizures, cerebral palsy, intellectual disability and other significant neurologic sequela [12,13,14]. An important area of clinical controversy is the treatment of mild HIE. While therapeutic hypothermia has been established as a standard of care for moderate-to-severe HIE, mild HIE cases were not included in the major hypothermia trials due to the perception of their favorable outcome. However, recent studies suggest that this population may also benefit from treatment [15]. Likewise, while data on neonates with severe acidemia (pH below 7.0 and BE ≤ −16) show a strong association with birth asphyxia and significant long-term adverse outcomes, the significance of mild-to-moderate acidemia is less clear. Recent studies suggest that even in a pH range of 7.00–7.19 there is increased risk for NICU admission and short-term neonatal morbidities including respiratory distress, sepsis, and encephalopathy [16,17,18].

Mild–moderate cord blood acidemia is not rare [16]. One study evaluating over 14,000 term births with available cord blood pH found a rate of 6.8% cases with pH between 7.0 and 7.2 [19]. However, there is no consensus or guidelines on the extent of clinical assessment, monitoring, laboratory tests and follow-up of these infants, nor information about the cost-effectiveness of these practices.

As there is no definite cutoff for abnormal cord pH, we employed a local policy in our unit in which infants with cord pH < 7.00 were admitted to NICU even if they were asymptomatic, and infants with cord pH ≥ 7.00 were assessed clinically in the well-baby nursery, with further workup and lab tests conducted at the discretion of the attending physician.

This study aimed to investigate short-term outcomes of late preterm and term infants born with mild- (pH range 7.13–7.20, base excess > −12.0) to-moderate (pH range 7.00–7.12, base excess ≤ −12) cord blood acidosis, and to compare the predictive ability of pH and base excess (BE). We included only term and late preterm infants as late preterms, who comprise a significant percentage of all preterm births, have a lower incidence of prematurity-related complications compared to younger preterm infants, minimizing confounding effects on the short-term outcomes we aimed to study.

## 2. Materials and Methods

This was a retrospective cohort study, which included term and late preterm (≥34 weeks) neonates born at Bnai Zion Medical Center in the years 2017–2022 with mild–moderate umbilical cord blood acidosis, defined as pH 7.0–7.2 or base excess −12 to −16. The cutoff value of base excess has been previously used by ACOG, and the pH range was used by other large-scale studies [17,18,20]. We further defined the acidosis type as “respiratory acidosis” when PCO_2_ was ≥60 mm Hg, “metabolic acidosis” when BE was ≤−8.0, and “mixed acidosis” when these two conditions co-existed. It is important to note that this definition was not derived from a pre-existing definition or empirical evidence but was decided for practical reasons based on previously published data of each parameter. Umbilical cord PCO_2_ ≥ 60 mm Hg was previously shown to correlate with neonatal morbidity [21], and BE ≤ −8 was shown to be associated with increased frequency of complications compared to the −4 to −8 range [22].

### 2.1. Cord Blood Sampling

Our local policy is that cord acid-base analysis is performed by the attending midwife in all instrumental deliveries, caesarian sections, cases requiring neonatal resuscitation, and in cases of significant or prolonged non-reassuring fetal heart rate, even if resuscitation is not required. Immediately after delivery, the cord is double clamped, and the blood sampling is carried out within 10 min, preferably from an artery; if arterial sampling is unsuccessful, a venous sample is obtained.

### 2.2. Neonatal Follow-Up

During the years 2017 and 2022, our department followed a local protocol in which infants born with cord pH ≥ 7.00 were followed in the well-baby nursery even if they had mild–moderate acidosis (pH 7.0–7.2) or base excess (BE) less than −12. In these cases, close clinical observation with physical examination, vital signs, and neurological assessment including modified Sarnat Score were performed and documented as soon as possible and up to 6 h of age, as well as careful charting of urine and stools for at least 48 h. Quantitative urine output was measured by diaper weight when urine appeared scant. Further blood tests, such as repeat blood gas and glucose within one hour of delivery and complete blood count (CBC), renal function tests (urea, creatinine) and liver enzymes (GOT/AST and GPT/ALT) at 24 h of life, were obtained at the discretion of the attending physician.

### 2.3. Data Extraction

Electronic medical records of all infants with pH levels ranging from 7.0 to 7.2 or base excess (BE) less than −12 born between 1 September 2017 and 28 February 2022 in our center were identified, and data including demographic, clinical, and laboratory measures were extracted. Clinical data included Apgar score, need for any or advanced resuscitation at birth defined as need for intubation with or without chest compression and medication, admission to neonatal intensive care unit (NICU), occurrence of signs of hypoxic ischemic encephalopathy (HIE), need for therapeutic hypothermia, respiratory distress, meconium aspiration syndrome, mechanical ventilation, sepsis, delayed initiation of feeding beyond 12 h after birth, length of hospital stay, and death. Biochemical measures included additional blood gases, CBC, renal and liver function tests if these were performed. AST levels ≥ 100 and creatinine levels > 1.0 were considered elevated based on previous studies [22,23]. The medical records of all cases of NICU admissions were carefully reviewed to determine the primary reason for admission and to verify the occurrence of the clinical outcomes coded by the ICD-10.

The study was approved by the institutional review ethics board, approval number 0055-22BNZ. Since data were retrospectively collected and anonymized, requirement for parental informed consent was waived.

### 2.4. Statistical Analysis

The cohort population was stratified based on the level of acidosis, mild or moderate, with separate analyses performed by definitions of pH and BE. Stratifying by pH, mild acidosis was defined as cord blood range pH 7.13–7.20 and moderate acidosis was defined as cord blood range pH 7.00–7.12. As there is no single universally accepted definition for mild or moderate acidosis, we selected this cutoff which approximately represents the 25th percentile of our sample. Stratifying by BE, mild acidosis was defined as BE > −12, and moderate acidosis as BE of −12 to −16 [5]. Data were statistically analyzed using SigmaPlot, version 11.0 (Systat Software Inc., San Jose, CA, USA) and Minitab^®^, version 16.2.2 (Minitab Inc., State College, PA, USA & Coventry, UK). All data were tested for normal distribution (Kolmogorov–Smirnov test). Statistical analysis included descriptive statistics and comparisons of parameters between the different cord blood pH or BE groups. For comparison of continuous variables between the groups with normal or non-parametric distributions, we used one-way analysis of variance (ANOVA) or Kruskal–Wallis one-way analysis of variance on ranks when normality test failed. For comparison of categorical data, chi-square analysis or Fisher’s exact test were used as appropriate. In order to study the prediction of cutoff values for possible adverse neonatal outcomes (AST ≥ 100 for liver and creatinine ≥ 1.1 for kidney function tests at 24 h) using pH or BE in cord blood gas, receiver operator curves (ROC) were plotted.

## 3. Results

### 3.1. Newborn Baseline Parameters and Cord Blood Characteristics

There were 13,800 births > 34 weeks of gestation during the study period. Three hundred and thirty-seven newborns were included in the study, 131 (38.8%) were female. The mean gestational age was 39.4 ± 1.5 weeks (range 34.0–41.6 weeks), and the mean birth weight was 3272 ± 479 g (range 1955–4760 g). Seventeen infants were late preterm, and the others were born ≥ 37 weeks of gestation. Apgar score at 1 min was <5 in 35 infants (10.3%) but none was <5 at 5 min, and only two infants required intubation at delivery. None required chest compressions or drug administration in the delivery room.

The infants were stratified separately by pH and by BE. Stratifying by pH, there were 86 infants in the moderate acidosis group (pH 7.0–7.12), and 251 infants in the mild acidosis group (pH 7.13–7.20). Stratifying by base excess (BE) levels, there were 90 infants in the moderate acidosis (BE −12 to −16) group, and 247 infants in the mild acidosis group (BE > −12). When applying both classifications, 218 infants had mild acidosis by both categories (pH and BE), and 57 infants had moderate acidosis by both. There were 33 infants whose moderate acidosis by base excess (BE −12 to −16) differed from their mild acidosis by pH (7.13–7.20), while 29 infants exhibited the opposite pattern: moderate acidosis by pH (7.00–7.12) but mild acidosis by base excess (BE > −12). Patient and cord gas characteristics by group are presented in Table 1. Gestational age was slightly higher in moderate acidosis compared to mild acidosis when stratified by base excess. Of the late preterm infants, only two had moderate acidosis, and the rest had mild acidosis. Apgar score at 1 and 5 min were significantly lower in the moderate acidosis group compared to the mild acidosis group in both stratifications. As noted, in this study we defined the acidosis type as respiratory acidosis when PCO2 was ≥60 mm Hg, as metabolic acidosis when BE was ≤−8.0, and as mixed acidosis when these two co-existed. By this definition, almost all cases of moderate acidosis (98%) were classified as metabolic or mixed acidosis, while in mild acidosis some of the cases (up to 18%) were classified as respiratory acidosis.

### 3.2. Clinical Outcomes

There were 48 infants admitted to NICU; however, 5 of them were admitted only due to late prematurity with no other medical issues, so that the admission rate for reasons other than prematurity was 12.8% (43/337). In 28 infants (8.3%), the primary reason for admission was respiratory distress. One infant required mechanical ventilation (0.3%) due to acute pulmonary hypertension requiring nitric oxide treatment, and 21 (6.2%) infants required non-invasive respiratory support. Of the infants requiring non-invasive ventilation: two were diagnosed with mild meconium aspiration syndrome by clinical signs and X-ray findings, and one had a pneumothorax. One infant requiring non-invasive respiratory support was diagnosed with mild hypoxic ischemic encephalopathy (stage 1 according to the modified Sarnat score) by clinical signs and amplitude integrated EEG and did not meet the criteria for therapeutic hypothermia. No other infant in our cohort showed any abnormal neurologic signs. One infant with moderate acidosis was admitted to NICU due to clinical signs of dehydration and failure to pass urine after 24 h of age and received intravenous fluids, though renal function tests were normal. Two asymptomatic infants were admitted to NICU due to significant hyponatremia (Na < 130 mmol/L) discovered through the bloodwork drawn due to moderate acidosis. Three were admitted for hypoglycemia related to gestational diabetes. One infant with mild cord blood acidosis presented at 14 h of life with clinical deterioration and was found to have a subdural hemorrhage. Other causes for admissions were workups and observation for suspected sepsis, congenital heart disease, and upper GI obstruction, which were all ruled out. Only one infant had delayed initiation of enteral feeding, which was started after 12 h. There were no deaths.

Infants with moderate acidosis had significantly more admissions to NICU, respiratory distress and need for respiratory support, and longer lengths of stay compared to infants with mild acidosis (Table 2 and Table 3).

### 3.3. Biochemical Measures

In 179 infants, repeated venous blood gas was obtained within one hour, and 44 infants had additional blood gas drawn after 2 h, and every hour until “normalization”. In those infants, the interval from birth (in hours) until the first blood gas measurement showing a pH ≥ 7.25 was charted.

Complete blood count (CBC) was performed at 24 h of age in 192 infants; 9 had mild thrombocytopenia (132,000–149,000), 12 had mild anemia (hematocrit range 40.7–44.9%), and 21 had polycythemia (hematocrit range 65–73.4%). In total, 13 infants had WBC count < 10,000 (range 1200–9700) and 8 had WBC ≥ 30,000 (30,000–39,300).

Renal function tests with or without electrolytes were taken at 24 h of age in 195 infants. Elevated creatinine levels above 1.0 mg/dL (range 1.1–1.5) were observed in 21 infants (21/195, 10.8%), with 4 cases in the mild acidosis group and 17 in the moderate acidosis group. None of the infants with elevated creatinine were late preterm. Creatinine levels > 1.1 mg/dL (range 1.2–1.5) were identified in nine infants (9/195, 4.6%), of whom seven had moderate acidosis and two had mild. Maternal creatinine levels at delivery were available for seven of these infants and were normal (range 0.6–1.0, mean 0.74 ± 0.14). Notably, five of these nine infants with creatinine > 1.1 mg/dL were asymptomatic and were identified solely through bloodwork performed due to moderate acidosis. The remaining four infants presented with respiratory distress and likely would have been detected through routine bloodwork upon NICU admission. In all cases, elevated creatinine was transient, although some of the infants received intravenous fluids.

Liver function tests at 24 h of life were conducted in some of the infants; AST was taken most often (in 194 infants). Elevated AST above 100 U/L was found in 12 newborns (12/194, 6.1%), 7 of whom were asymptomatic and 5 were admitted to NICU for other reasons, and elevated ALT >50 U/L was found in 3 infants. In five cases (5/194, 2.5%), both creatinine and AST levels were abnormally elevated.

The results of the biochemical measures by base excess stratification and by pH stratification are presented in Table 2 and Table 3, respectively. Infants with moderate acidosis took longer to improve compared to mild acidosis. Infants with moderate acidosis had significantly lower hematocrit, higher creatinine and higher AST compared to mild acidosis, in both stratifications. However, levels of ALT, GGT and ALK-P were lower in moderate acidosis compared to mild. None of the statistically significant differences between the groups in laboratory values were clinically meaningful.

### 3.4. ROC Analysis

**Figure 1:**
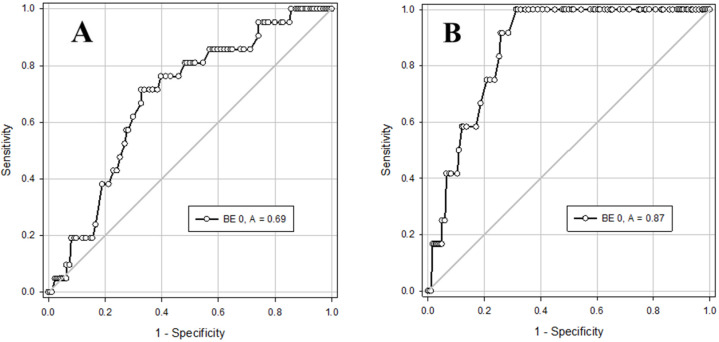
ROC Curves and cutoff values of cord blood gas BE to predict infants who are likely to have abnormal kidney or liver function test. (**A**) for identifying creatinine ≥ 1.1: area under curve (AUC) = 0.69 [95% confidence interval (CI) 0.58–0.79], *p* < 0.005. Using BE cutoff ≤ −12 the sensitivity is 0.71 (95% CI 0.64–0.78) and the specificity is 0.67 (95% CI 0.60–0.74); (**B**) for identifying AST ≥ 100: AUC = 0.87 [95% CI 0.80–0.93], *p* < 0.001. Using BE cutoff ≤ −12 the sensitivity is 1.00 (95% CI 0.98–1.00) and the specificity is 0.69 (95% CI 0.61–0.75).

**Figure 2:**
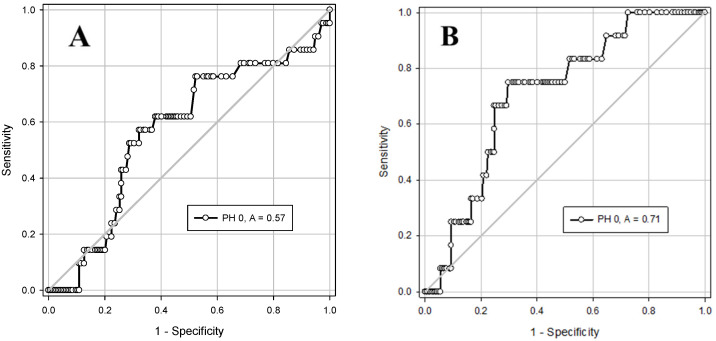
ROC curves and cutoff values of cord blood gas pH to predict infants who are likely to have abnormal kidney or liver function test. (**A**) for identifying creatinine ≥ 1.1: area under curve (AUC) = 0.57 [95% confidence interval (CI) 0.44–0.70], *p* = 0.2900. Using pH cutoff ≤ 7.12 the sensitivity is 0.52 (95% CI 0.44–0.60) and the specificity is 0.71 (95% CI 0.63–0.77); (**B**) for identifying AST ≥ 100: AUC = 0.71 [95% CI 0.58–0.83], *p* = 0.01. Using pH cutoff ≤ 7.12 the sensitivity is 0.75 (95% CI 0.67–0.81) and the specificity is 0.70 (95% CI 0.63–0.76).

Comparing the predictive ability of pH and base excess (BE) in cord blood gas to identify infants with creatinine ≥ 1.1 or AST ≥ 100 at 24 h of age, using ROC curves, BE ≤ −12 was found to be a better predictor than pH ≤ 7.12 (Figure 1 and Figure 2).

## 4. Discussion

Information obtained from umbilical cord gas is valuable for assessing the newborn’s past, present and future condition. It may serve as a marker of peripartum or intrapartum hypoxia, guide prompt treatment of acidemia, provide prognostic information on the newborn and may be important in cases of litigation.

This study retrospectively evaluated short-term outcomes of mild–moderate cord blood acidemia obtained from high-risk deliveries. We found a rate of 12.8% of NICU admissions in this population, rising to 27% among newborns with moderate acidosis. Likewise, in a study that performed universal cord gas sampling in all deliveries, NICU admission rate of term infants reached 23% in a similar pH range of moderate acidosis [24]. This rate is substantially higher than the rate of 2.8% of NICU admission rate reported in the general term infant population [25]. Most NICU admissions in our study were due to respiratory distress and need for non-invasive respiratory support, and only two had serious medical conditions. These findings are consistent with previous studies that showed that even mild-to-moderate acidosis increases the risk of respiratory distress, meconium aspiration syndrome and sepsis [17,20,24,26]. Namely, a recent study by Bailey et al. suggested an increase in morbidities such as respiratory distress and sepsis as pH decreases in infants exhibiting mild umbilical artery acidosis, i.e., pH 7.11–7.19 when compared to those with umbilical artery pH ≥ 7.20 [17]. In a large population-based cohort study, Andersson et al. showed an increased risk for respiratory support in infants with cord pH of 7.00–7.09 and to a lesser degree in those with cord pH 7.10–7.19 compared to infants with cord pH > 7.20 [18]. Sabol et al. reported similar findings in infants with reassuring 5 min Apgar scores ≥ 7 [20], and Bligard et al. demonstrated increased risk of adverse neonatal outcome with mild cord blood acidemia even in newborns born by a scheduled caesarian delivery with reassuring preoperative monitoring [26]. Notably, a large-scale study in the general population of term infants found that 47% of NICU admissions were due to respiratory disorders [25]. Collectively, these findings emphasize the significance of clinically monitoring and identifying this patient population. None of the infants in our study had signs of moderate–severe HIE necessitating therapeutic hypothermia, which is consistent with most previous studies reporting that the risk for HIE rises substantially only below pH 7.00 [27]. Studies on long-term neurodevelopmental outcome of well appearing infants with mild–moderate acidosis are limited and suggest that there is no increased risk for abnormal outcome in infants without signs of encephalopathy [28,29].

Multi-organ dysfunction after fetal hypoxia is thought to be related to the diving reflex, which involves shunting blood away from the skin and splanchnic circulation toward the heart, adrenals, and brain. This adaptive response is thought to protect these vital organs from hypoxic-ischemic injury, and if activated long enough may cause dysfunction of non-essential organs such as liver and kidney [30]. Studies on infants with severe HIE and evidence of acute brain injury have reported high rates of multi-organ failure, which can include renal, hepatic, hematologic, cardiac and gastrointestinal injury [23,31]. However, the severity of neurologic injury does not always correlate with the extent of injury to other organs [30], and injury may occur even in the absence of significant neurologic symptoms [32]. Interestingly, one study of confirmed perinatal asphyxia cases [33] found that creatinine values were higher in a partial prolonged pattern of asphyxia injury than in the acute profound pattern. This suggests that sustained less severe hypoxia may lead to more significant injury in the kidneys compared to a sudden severe insult. Based on these findings, the authors recommended routine newborn laboratory investigations, including creatinine, for all near-term and term newborns with low 5 min Apgar and a cord base excess ≤ −12 to monitor for multi-organ failure. Only limited data is available on multi-organ involvement in newborns with mild–moderate acidemia at birth. In one study [32], outcomes were reported for 65 term newborns with none to mild HIE, 40 of whom had moderate acidosis (pH range 7.00–7.10). Among these infants, renal dysfunction was observed in 7.5%, liver dysfunction in 17.5% and hematologic abnormalities in 7.5%. In our study, creatinine at 24 h of life was significantly higher in moderate acidosis compared to mild acidosis, and was significantly elevated in nine cases, some of whom received intravenous fluids. In most of them, this was only detected through bloodwork conducted due to the presence of cord blood acidemia with no other symptoms to alert the clinician. This finding may indicate that there had been some degree of perinatal insult affecting the kidney in these cases, as was previously described in asphyxiated newborns. Another biomarker found to be in correlation with the extent of the hypoxic ischemic insult is AST, which reflected hepatic injury in previous studies. In a study of moderate and severe HIE cases [34], AST as well as ALT differed according to HIE grade. Another study comparing term infants with and without perinatal asphyxia found that AST level in the first day of life correlated well with the severity of HIE, and this was an early diagnostic marker [35]. In our study, AST was significantly elevated in moderate compared to mild acidosis, suggesting that liver involvement may occur even in perinatal insults not associated with the complete HIE clinical picture. Hematocrit was slightly lower in moderate acidosis compared to mild in our study, in agreement with some previous studies showing lower hematocrits in asphyxiated newborns [36,37], possibly due to oxidant stress. However, the differences were not clinically meaningful and none of the infants in our cohort was severely anemic. Our findings of certain biochemical abnormalities suggest some impact of moderate acidosis on bone marrow, liver, and kidney function, even in well-appearing infants, and while in the majority of cases in our cohort it had minimal clinical significance, a few of the cases were important to detect and warranted clinical attention. This was particularly evident in infants who, despite being otherwise asymptomatic with reassuring Apgar scores and normal urine output, exhibited significantly elevated creatinine on bloodwork that prompted the administration of intravenous fluids. Therefore, we suggest monitoring renal function in all infants with moderate acidosis. While the observed liver function and hematologic abnormalities were subtle and did not require any intervention, they may serve as indicators of a more substantial perinatal insult.

We found that base excess was a more reliable predictor than pH of elevated AST and elevated creatinine, which were chosen as markers of perinatal insult [34]. This is physiologically plausible. While pH directly measures the hydrogen ion concentration in the blood, and does not distinguish between respiratory and metabolic causes of acid-base disturbances, base excess is calculated using algorithms incorporating pH and PaCO_2_ or bicarbonate (HCO_3_**^−^**) and reflects the metabolic component of the acid base status [38]. Furthermore, because pH represents the inverse log of the hydrogen ion concentration, it changes exponentially rather than linearly, and may not reflect the “linear” nature of the accumulation of acid proportionate to the hypoxemic ischemic insult. In contrast, base excess reflects the excess or deficit of base buffer and therefore has a linear and not logarithmic correlation to the degree of acidosis [38]. Thus, it is likely that base excess is better correlated with true substantial fetal distress. Supporting our finding, a large study [39] prospectively collecting universal blood gas and lactate in all deliveries found that both umbilical cord lactate and base excess were more sensitive and specific for predicting neonatal morbidity than pH, with similar areas under the ROC curve. The authors noted that lactate has the advantage of being measured directly rather than calculated.

Although the risk for adverse outcomes progresses as a continuum with worsening cord pH or base excess values, establishing an operational threshold is helpful in identifying newborns who need closer observation and follow-up, as the likelihood of adverse outcomes increases below this point. The pH threshold for predicting unfavorable short-term outcomes in neonates has been previously studied but no definite threshold value was defined. From a neurological standpoint, a cutoff of 7.00 has been the standard to select infants at high risk for hypoxic-ischemic encephalopathy [5] and often serves as one of the criteria used for starting therapeutic hypothermia [27]. However, it is possible that a higher threshold is required to capture dysfunction in other organs that can appear in neurologically normal infants exposed to a lesser degree of fetal distress. While some studies [24,40] supported the use of umbilical pH cutoff of 7.2 as the predictor for negative clinical outcomes, others recommended a cutoff of 7.11 [17] or 7.10 [16]. Our data suggest that neonates who present with umbilical cord pH ≤ 7.12, or more specifically BE values ≤ −12 may require closer observation. Below this threshold the rate of NICU admissions increases, and clinically asymptomatic renal or liver dysfunction may be detected and monitored.

The main strength of our study is providing data on multiple clinical and biochemical measures in infants born with mild–moderate cord blood acidemia, a group that has been relatively understudied compared to infants with more severe acidemia. However, this study also has certain limitations. First, its retrospective nature and absence of universal cord gas screening in our center resulted in the inclusion of mostly high-risk deliveries in which cord blood gas was obtained by protocol. Consequently, we have no data of cord blood gas from normal uneventful deliveries, some of which may also have had acidemia. In addition, we did not conduct long-term follow-up as it was beyond the scope of this study and, therefore, we cannot provide evidence regarding neurodevelopment, renal or liver function in infancy. Furthermore, we could not control for all possible confounders, such as maternal morbidities, and were limited to the information available from the records. Last, we did not have paired samples of arterial and venous cord blood and cannot be certain that they are all arterial, as occasional sampling difficulties sometimes lead to venous samples.

## 5. Conclusions

In conclusion, moderate acidosis in cord blood is associated with an increased incidence of neonatal morbidities, NICU admissions and renal or liver dysfunction compared to mild cord blood acidemia. BE was better correlated with short-term adverse outcomes than pH in this retrospective study. Well-appearing infants with cord gas BE levels < −12 may benefit from closer clinical monitoring of neurologic status, urine output and laboratory investigation of renal and liver function, even though most lab abnormalities were mild and did not require intervention. Future prospective studies, including long-term neurodevelopmental follow-up are needed to fully understand the clinical impact of moderate acidosis and BE thresholds.

## Figures and Tables

**Table 1:** Patient and cord gas characteristics by pH and base excess groups.

	pH 7.00–7.12 *n* = 86	pH 7.13–7.20 *n* = 251	*p*-Value
Gestational Age (weeks) *	39.6 (38.6, 40.5)	39.6 (38.6, 40.5)	0.867
Birth Weight (g) **	3260 ± 484	3276 ± 477	0.795
Female *n* (%)	39 (45)	92 (37)	0.194
Delivery Mode *n* (%)			0.478
Vaginal	22 (25)	50 (20)	
Vacuum	22 (25)	76 (30)	
Emergency C/S	36 (42)	114 (45)	
Elective C/S	6 (7)	11 (4)	
APGAR 1 min *	8 (6, 9)	8 (7, 9)	0.004
APGAR 5 min *	9 (9, 10)	9 (9, 10)	0.030
Cord pH *	7.09 (7.06, 7.11)	7.17 (7.15, 7.18)	<0.001
Cord Base excess **	−12.7 ± 1.8	−9.6 ± 1.9	<0.001
Cord Base excess ≤ −12 *n* (%)	57 (66)	33 (13)	<0.001
Cord HCO_3_ *	22.3 (20.6, 23.6)	23.1 (21.6, 24.5)	0.007
Cord pCO_2_ *	75.7 (70.2, 82.7)	65.4 (60.1, 69.4)	<0.001
Acidosis type *n* (%)			<0.001
Respiratory	1 (1)	44 (17)	
Metabolic	3 (3)	59 (23)	
Mixed	82 (95)	148 (58)	
	**BE −12 to −16** ***n* = 90**	**BE > −12** ***n* = 247**	***p*-Value**
Gestational Age (weeks)	39.9 (39.1, 40.7)	39.4 (38.5, 40.5)	0.027
Birth Weight (g) **	3281 ± 490	3268 ± 475	0.836
Female *n* (%)	35 (39)	96 (39)	0.902
Delivery Mode *n* (%)			0.019
Vaginal	25 (28)	47 (19)	
Vacuum	30 (33)	68 (28)	
Emergency C/S	28 (31)	122 (49)	
Elective C/S	7 (8)	10 (4)	
Apgar 1 *	8 (6, 9)	8 (7, 9)	0.002
Apgar 5 *	9 (8, 10)	9 (9, 10)	0.011
Cord pH *	7.10 (7.06, 7.14)	7.17 (7.14, 7.18)	<0.001
Cord pH ≤ 7.12 *n* (%)	57 (63)	29 (12)	<0.001
Cord Base excess *	−13.0 (−14.0, −12.5)	−9.4 (−10.6, −8.2)	<0.001
Cord HCO_3_ **	20.1 ± 2	23.6 ± 1.9	<0.001
Cord pCO_2_ *	66.3 (56.1, 75.9)	67.1 (36.2, 72.5)	0.220
Acidosis type *n* (%)			<0.001
Respiratory	0 (0)	45 (18)	
Metabolic	31 (34)	31 (13)	
Mixed	59 (65)	171 (69)	

* median (25%, 75% IQR); ** mean ± SD for continuous data.

**Table 2:** Clinical and laboratory outcomes by base excess.

	BE −12 to −16 *n* = 90	BE > −12 *n* = 247	*p*-Value
Clinical outcomes			
NICU admission *n* (%)	22 (24)	26 (11)	0.002
HIE any *n* (%)	1 (0.4)	0 (0)	0.598
Respiratory distress *n* (%)	16 (18)	12 (5)	<0.001
Meconium aspiration syndrome *n* (%)	1 (1)	1 (0.4)	0.956
Mechanical ventilation *n* (%)	1 (1)	0 (0)	0.598
Non-invasive respiratory support *n* (%)	12 (13)	9 (4)	0.003
Length of hospital stay *	4 (3, 5)	3 (3, 4)	<0.001
Laboratory measures			
Base excess after 1 h *	−7.9 (−10.2, −6.8) *n* = 69	−6.3 (−7.1, −4.9) *n* = 110	<0.001
Time to pH ≥ 7.25 h *	2.25 (1, 6) *n* = 66	2 (1, 3) *n* = 108	0.007
WBC * ×10	18 (14, 22) *n* = 74	17 (13, 21) *n* = 116	0.152
Hct ** %	52 ± 5.9 *n* = 72	57 ± 7.2 *n* = 118	<0.001
Plt * ×100	254 (213, 289) *n* = 73	242 (204, 287) *n* = 116	0.430
Urea * mg/dL	26 (21, 30) *n* = 72	25 (19, 29) *n* = 123	0.151
Creatinine * mg/dL	0.9 (0.7, 1.0) *n* = 72	0.8 (0.6, 0.9) *n* = 123	0.007
AST * U/L	64 (48, 80) *n* = 70	49 (38, 65) *n* = 124	<0.001
ALT * U/L	22 (13, 28) *n* = 71	26 (22, 34) *n* = 122	0.001
GGT * U/L	91 (67, 111) *n* = 62	98 (84, 123) *n* = 110	0.013
ALK-P * U/L	178 (141, 221) *n* = 63	198 (189, 222) *n* = 115	0.005

* median (IQR 25%, 75%); ** mean ± SD; HIE—hypoxic ischemic encephalopathy; WBC—white blood cells; Hct—hematocrit; Plt—platelets; AST—aspartate aminotransferase; ALT—alanine transaminase; GGT—gamma-glutamyl transpeptidase; ALK-P—alkaline phosphatase.

**Table 3:** Clinical and laboratory outcomes by pH.

	pH 7.00–7.12 *n* = 86	pH 7.13–7.20 *n* = 251	*p*-Value
Clinical outcomes			
NICU admission *n* (%)	23 (27)	25 (10)	<0.001
HIE any *n* (%)	1 (1)	0 (0)	0.574
Respiratory distress *n* (%)	15 (17)	13 (5)	<0.001
Meconium aspiration syndrome *n* (%)	2 (2)	0 (0)	0.107
Mechanical ventilation *n* (%)	1 (1)	0 (0)	0.574
Non-invasive respiratory support *n* (%)	12 (14)	9 (4)	0.002
Length of hospital stay *	4 (4, 5)	3 (3, 4)	<0.001
Laboratory measures			
pH after 1 h *	7.24 (7.13, 7.29) *n* = 48	7.33 (7.28, 7.36) *n* = 131	<0.001
Time to pH ≥ 7.25 h *	3.5 (1, 6.5) *n* = 48	2 (1, 3) *n* = 126	0.001
WBC * ×10	18.6 (13.6, 20.8) *n* = 59	17.0 (13.3, 22.1) *n* = 131	0.430
Hct ** %	54.1 ± 6.4 *n* = 60	56.3 ± 7.3 *n* = 132	0.040
Plt * ×100	252 (205, 282) *n* = 59	247 (211, 290) *n* = 130	0.866
Urea * mg/dL	26 (20, 32) *n* = 53	25 (20, 29) *n* = 142	0.280
Creatinine * mg/dL	0.8 (0.8, 1.0) *n* = 53	0.8 (0.7, 0.9) *n* = 142	0.039
AST * U/L	65 (45, 80) *n* = 52	53 (38, 66) *n* = 142	0.002
ALT * U/L	20 (13, 29) *n* = 51	26 (21, 33) *n* = 142	0.005
GGT * U/L	89 (66, 111) *n* = 40	98 (77, 122) *n* = 132	0.022
ALK-P * U/L	187 (141, 222) *n* = 44	198 (184, 221) *n* = 134	0.110

* median (IQR 25%, 75%); ** mean ± SD; HIE—hypoxic ischemic encephalopathy; WBC—white blood cells; Hct—hematocrit; Plt—platelets; AST—aspartate aminotransferase; ALT—alanine transaminase; GGT—gamma-glutamyl transpeptidase; ALK-P—alkaline phosphatase.

## Data Availability

Data are unavailable due to ethical restrictions made by the ethics committee.

## Abbreviations

The following abbreviations are used in this manuscript:

NICU	Neonatal intensive care unit
BE	Base excess
HIE	Hypoxic ischemic encephalopathy
ROC	Receiver operator curve
